# Application Status and Prospects of Artificial Intelligence in Peptic Ulcers

**DOI:** 10.3389/fsurg.2022.894775

**Published:** 2022-06-16

**Authors:** Peng-yue Zhao, Ke Han, Ren-qi Yao, Chao Ren, Xiao-hui Du

**Affiliations:** ^1^ Department of General Surgery, First Medical Center of the Chinese PLA General Hospital, Beijing, China; ^2^ Department of Gastroenterology, First Medical Center of the Chinese PLA General Hospital, Beijing, China; ^3^ Translational Medicine Research Center, Medical Innovation Research Division and Fourth Medical Center of the Chinese PLA General Hospital, Beijing, China; ^4^ Department of Pulmonary and Critical Care Medicine, Beijing Chaoyang Hospital, Capital Medical University, Beijing, China

**Keywords:** peptic ulcer, artificial intelligence, gastric ulcer, complications, convolutional neural network

## Abstract

Peptic ulcer (PU) is a common and frequently occurring disease. Although PU seriously threatens the lives and health of global residents, the applications of artificial intelligence (AI) have strongly promoted diversification and modernization in the diagnosis and treatment of PU. This minireview elaborates on the research progress of AI in the field of PU, from PU’s pathogenic factor *Helicobacter pylori (Hp)* infection, diagnosis and differential diagnosis, to its management and complications (bleeding, obstruction, perforation and canceration). Finally, the challenges and prospects of AI application in PU are prospected and expounded. With the in-depth understanding of modern medical technology, AI remains a promising option in the management of PU patients and plays a more indispensable role. How to realize the robustness, versatility and diversity of multifunctional AI systems in PU and conduct multicenter prospective clinical research as soon as possible are the top priorities in the future.

## Introduction

Peptic ulcer (PU) is an inflammatory reaction and necrotizing lesion of the mucosa or submucosa under the action of various pathogenic factors. It often occurs in the gastrointestinal mucosa that performs the function of gastric acid secretion, of which the stomach and duodenum are the most common. It is estimated that the incidence of PU is 0.1%–0.3% per year, while its lifetime prevalence reaches as high as 5%–10% in the general population (1). Therefore, early diagnosis and prevention of PU is crucial to reduce the economic burden on global health.

Artificial intelligence (AI) refers to a modern technology that imitates human behavior and thinking through computer networks and is an interdisciplinary subject developed on the basis of computer science, information theory, determinism, neuropsychology, philosophy, linguistics, etc. (2). With the advent of the era of big data, AI has achieved rapid development in the field of image and speech recognition with the help of technological innovations such as machine learning (ML) and deep learning (DL). Among them, the most advanced and common one is the DL technology represented by convolutional neural network (CNN), which is currently widely used in many fields (3).

The concept of DL is inspired by the synaptic system of the human brain’s neural network. It is composed of multiple layers of simple computing nodes that simulate the activities of the human visual cortex through complex connections. In terms of specific research content, DL mainly includes CNNs, self-coding neural networks and deep belief networks (DBNs) (4). DL can identify important features from a large database of images through a repeated learning process. The larger the data volume given to it, the more obvious the advantages of DL, namely, the faster and higher accuracy of recognition. In the medical field, DL-based intelligent systems can automatically extract and learn clinical data, which can not only help doctors diagnose diseases but also accurately predict prognosis. Currently, DL has been prominent in the diagnosis of lung cancer, breast cancer, brain cancer, prostate cancer, Alzheimer's disease and Parkinson's disease, and has also been widely reported in the clinical diagnosis and treatment of digestive diseases (5, 6).

**Figure 1:**
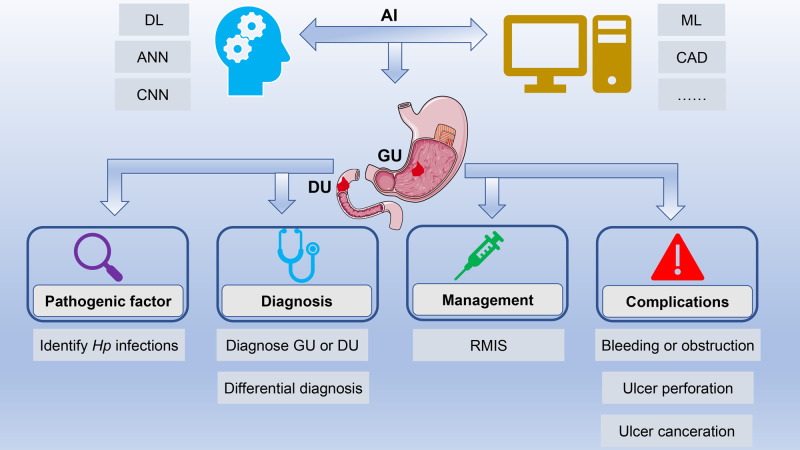
Application of artificial intelligence in peptic ulcers. AI has achieved rapid development in the field of PU with the help of technological innovations such as ML, DL, and CNN. AI is widely applied in the field of PU, ranging from its pathogenic factors, diagnosis and differential diagnosis to management and complications. Abbreviations: AI, artificial intelligence; PU, peptic ulcer; ML, machine learning; DL, deep learning; CNN, convolutional neural network.

The differential diagnosis of benign and malignant ulcers of the digestive tract is significantly important for subsequent treatment. However, macroscopic endoscopic diagnosis is sometimes very burdensome, since the accuracy of endoscopic diagnosis largely depends on the technical level and clinical experience of endoscopists. Moreover, massive image data also require considerable time and efforts. Fortunately, the emergence of AI can solve the above problems. This minireview elaborated on the research progress of AI in the field of PU, from its pathogenic factors, diagnosis, to management and complications (Figure 1). Moreover, the challenges and prospects in this field were also elaborated.

## Application of AI in PU

### AI in the Pathogenic Factors of PU

*Helicobacter pylori (Hp)* infection is one of the important pathogenic factors of PU. Statistics show that the positive rates of *Hp* in gastric ulcer (GU) and duodenal ulcer (DU) patients are 60%–80% and 90%, respectively (7, 8). In terms of pathogenesis, on the one hand, *Hp* can release urease to break down urea to produce NH_3_, which destroys the acidic environment of the gastrointestinal tract. However, *Hp* can generate numerous toxin proteins that destroy the barrier system of the gastric mucosa. Therefore, early identification of *Hp* infection is essential in preventing PU. At present, noninvasive tests, such as urea breath and stool antigen tests, are still the first choice for the exclusion of *Hp* infection, while elderly people over 60 can select direct gastroscopy for exclusion (9). However, most cases of subclinical *Hp* infection still rely on invasive biopsy, which takes time to avoid misdiagnosis. In addition, the severity of *Hp* infection needs to be measured by the inspector with the naked eye. This method is a subjective judgment, and there will inevitably be bias. Fortunately, the emergence of AI may shed light on the current dilemma.

The earliest application of AI technology in *Hp* infection recognition was in 2004. Huang et al. (10) first trained AI by using endoscopic images of 30 dyspeptic patients (15 *Hp* infections and 15 non-*Hp* infections) and established a refined feature selection with neural network (RFSNN) algorithm. Then, a verification test was performed on the images of the remaining 74 patients with dyspepsia. The sensitivity, specificity, and accuracy of the algorithm for identifying *Hp* infection reached 78.8%, 90.2%, and 85.1%, respectively. Since then, AI technology has developed rapidly, and CNNs have also emerged and quickly become an absolute leader in the field of medical image processing. In 2017, Shichijo et al. (11) adopted 32,208 photos from 1750 patients (735 *Hp* positive and 1,015 *Hp* negative) as a discovery cohort to train a CNN model, followed by validation of its diagnostic performance in an independent dataset. Finally, it was verified on a new data set to compare the difference between CNN and endoscopists in identifying *Hp* infections. The results showed that the sensitivity, specificity, accuracy, and diagnosis time of the CNN were 88.9%, 87.4%, 87.7% and 194 s, respectively, and these indicators were 79.0%, 83.2%, 82.4%, and 230 ± 65 min among 23 endoscopists. This showed that using a CNN to diagnose *Hp* infection has higher accuracy and costs less time than manual diagnosis by endoscopists.

There are many similar studies on AI in predicting *Hp* infection (12–16), all of which demonstrate AI’s superior accuracy and sensitivity (Supplementary Table S1). However, AI’s ability to recognize *Hp* infection still has some limitations that need to be overcome in the future. For example, the training and test sets of the above studies were all from one medical center or one country, and more continuous and rigorous external validation from various sources is necessary to ensure the credibility of conclusions.

### AI in the Diagnosis and Differential Diagnosis of PU

Typical GU is more common in the gastric angle or lesser curvature. The lesions are mostly round or oval and generally solitary but can also be multiple. Most benign GUs are small in diameter and have regular edges. The surrounding mucosa often has hyperemia and edema, and the surface is mostly covered with white or yellow exudate. The morphology of DU is similar to that of GU. DU mostly occurs in the duodenal bulb, especially near the anterior or posterior wall of the gastric pylorus. Under normal circumstances, gastroscopy physicians can make corresponding clinical diagnoses based on the subject’s gastrointestinal morphology or histological abnormalities, but if the situation is more complicated or the endoscopists are not fully sure, then they will take some specimens from around the lesion for pathological examination and give the final pathological diagnosis.

However, gastroscopy has two limitations: first, the sensitivity and specificity of diagnosis are closely related to the level of the examiner; if the examiner lacks clinical experience, misdiagnosis and missed diagnosis of PU can easily occur. Second, gastroenterologists need to check the abnormalities of numerous images or videos to detect the patient’s lesions. However, considering the limited time and energy of clinicians, such a large number of images or videos will undoubtedly increase their workload.

The application of AI in PU diagnosis can be traced back to 2002. Saenz Bajo et al. (17) used the Neurone network to differentiate between PU and functional or idiopathic dyspepsia on the basis of clinical notes. The researchers classified and verified 81 patients who were clinically diagnosed with dyspepsia according to the presence of determined symptoms and finally found that the Neurone network successfully classified 81% of patients with negative and positive predictive values of 90% and 80%, respectively. To improve the diagnostic efficiency of GU, Al-Kasasbeh et al. (18) constructed a fuzzy logic decision-making system based on the variations in electrical resistance of acupuncture points, and the result was encouraging. The prediction error level of this decision-making system was not higher than 0.18, which once again proved the feasibility of AI application in PU.

In the past three years, with the increasingly widespread application of DL and CNN in the medical field, a body of researches on AI in PU diagnosis and differential diagnosis have emerged. To explore the ability of deep CNN to identify ulcers in wireless capsule endoscopic images, Wang et al. (19) first used 15,781 ulcer frames to train deep CNN, then used 2040 ulcer and 2,319 normal frames for verification, and finally performed it on 4,917 ulcer and 5,007 normal frames for testing. The results showed that the overall sensitivity of deep CNN in diagnosing ulcers was 89.7%, and the overall specificity and accuracy were both higher than 90%. Similarly, Alaskar et al. (20) built a CNN to detect its effect in diagnosing gastrointestinal ulcers. They trained and tested 336 and 105 photos respectively. Finally, the sensitivity, specificity and accuracy of the CNN in diagnosing gastrointestinal ulcers all reached an astonishing 100%.

In addition to its extraordinary performance in the diagnosis of PU, AI also plays an indispensable role in the discrimination of PU from other gastrointestinal diseases. To differentially diagnose the two most common stomach deformities (ulcers and bleeding), Khan et al. (21) constructed a rank-based deep features selection system that was verified by 4,000 video frames of ulcers, 4,000 video frames of bleeding and 4,000 normal ones, and found that the system only took 21.15 sec to identify all these video frames with an accuracy of 99.5%. This will undoubtedly greatly improve the work efficiency of gastroenterology clinicians. Majid et al. (22) and Xia et al. (23) established a CNN model to explore its differential diagnostic ability of four types of stomach infections (ulcer, polyp, esophagitis, and bleeding) and seven types of gastric lesions (erosion, polyp, ulcer, submucosal tumor, xanthoma, normal mucosa, and bleeding), respectively. The research results all proved the excellent differential diagnosis ability of CNN. The accuracy of the former reached 96.5%, while the sensitivity, specificity and accuracy of the latter were 96.2%, 76.2% and 77.1%, respectively. There are many similar studies (24–26), and detailed information can be seen in Supplementary Table S2.

### AI in the Management of PU

In addition to ML and CNN, robots are also an outstanding representative of the development and application of AI in medicine. For example, the Da Vinci robot has played a crucial and irreplaceable role in multiple surgical disciplines, becoming the most representative achievement of minimally invasive surgery and intelligent medicine. The progress of robots in the field of PU is relatively slow, mainly because the proportion of surgical intervention in the treatment of PU is inherently small.

Sutures are one of the most difficult tasks in robot-assisted minimally invasive surgery (RMIS), because the surgeon needs to coordinate and control three or four tools, which will distract and consume the surgeon’s energy and prolong the operation time. Gao et al. (27) proposed a robot autonomous suture task allocation method, and conducted a suture repair experiment for DU under the guidance of a surgeon. The results showed that this method obtained an optimal suture task allocation plan, which was beneficial to improve the intelligent degree of robot operation. Brungardt et al. (28) explored the feasibility of right-side robot-assisted transthoracic vagotomy for the treatment of marginal ulcers after gastric bypass surgery for the first time. The patient was a 43-year-old white female who underwent Roux-en-Y gastric bypass surgery at the age of 29. The author’s team successfully ligated two vagus nerves through right-side robot-assisted thoracoscopic surgery, and the patient had a good prognosis. This successful case provides a new idea for expanding the application of robotic surgery in highly selective transabdominal vagotomy.

### AI in the Complications of PU

#### Bleeding

When the mucosal damage of peptic ulcer has exceeded the basal layer of the mucosa, if it is further deepened, it may impair the blood vessels under the mucosa and cause bleeding. Bleeding in the digestive tract is the most common complication of PU. Nearly 20% of ulcer-related bleeding cases had no obvious alarm symptoms or signs before onset. Early recognition of the risk of bleeding and its related adverse outcomes can help doctors provide timely intervention, which may improve the prognosis.

Traditional prediction methods based on electronic health records usually do not take the correlation between static and dynamic data into consideration, but these data contain important information about the interaction effects which are important to fit the association between clinical materials and outcomes. Tan et al. paid attention to this point and developed a novel end-to-end importance perception personalized DL method (eiPDLA), which improved the accuracy of early bleeding risk at 1 year ahead with an AUC of 0.944 (29). The team also made relevant improvements in predicting mortality in patients with PU bleeding (30). The fatality rate of PU bleeding was greatly related to age, complications, severity of bleeding and recurrence of bleeding. Through the multiconvolution deep residual network (ResNet), deep fusion and long short-term memory (LSTM) methods, the AUC of the mortality prediction model for patients with PU bleeding reached 0.9353. ML models were also used to predict the risk of recurrent bleeding in idiopathic ulcers (31), which was characterized by occurring without *Hp* infection or the use of non-steroidal anti-inflammatory drugs (NSAIDs) and a high risk of recurrent bleeding and death. The idiopathic peptic ulcer ML (IPU-ML) model built in this study was trained by 22 854 patients with a diagnosis of PU disease and tested by 1,265 patients who were diagnosed with GI bleeding. It could identify patients who had 1-year recurrent ulcer bleeding, with an overall accuracy of 84.3% and an AUC of 0.775, especially idiopathic ulcer patients who were at low risk of recurrent ulcer bleeding.

AI also plays a role in guiding the practice of endoscopy. It could help identify the risk of PU bleeding under endoscopy according to the Forrest classification (32). After training on 2,378 static endoscopic images from 1,694 PU patients, the DL model had moderate to substantial consistency with advanced endoscopists on the test data set, which was higher than that of novice endoscopy. Therefore, this had certain application value for training young doctors and helping to make decisions in emergency endoscopy.

#### Perforation

PU perforation refers to the deep development of GU or DU, which penetrates the serosal layer and causes local flatulence and perforation. It is a severe clinical complication and a leading cause of operation-related death. Early identification of patients with perforated ulcers with poor prognosis is of great importance to patient risk stratification and identification of potential treatment. An artificial neural network (ANN) model was constructed to identify risk factors (increasing age, the presence of an active cancer, a delay from admission to surgery >24 h, hypoalbuminemia, hyperbilirubinemia and increasing creatinine values) of the 30-day mortality after surgery and their complex interactions with the mortality among patients with PU perforation (33). Among the 168 patients included in the study, the data of 117 patients were used to train the model, and 51 patients were used to test it. The mortality predicted by ANN showed an AUC of 0.90 (95% confidence interval [CI], 0.85–0.95). However, the study was restricted by its small sample size, and relied on predictor variables that were previously defined rather than extensive screening to build the model. Moreover, this research lacked true, secondary and external verification queues. Therefore, it is still necessary to enhance the accuracy of prediction to generate a more reliable model for future risk projection and clinical decision making of PU treatment.

Karargyris’s team (34) developed a wireless capsule endoscope that could identify small intestinal perforated ulcers and polyps, but no similar identification method had been seen to assist in identifying PU perforations in endoscopy.

#### Pyloric Obstruction

Obstruction is often seen in DU and pyloric duct ulcers. The pylorus is the narrowest part of the digestive tract, with a normal diameter of approximately 1.5 cm, so it is prone to obstruction. PU may cause inflammation and swelling of the tissues around the pylorus, leading to obstruction. Temporary obstruction can be resolved after the ulcer has healed. However, pyloric ulcer scars can also cause intractable mechanical obstruction, which requires an endoscopic or surgical operation to relieve the obstruction. Unfortunately, to our knowledge, there is no relevant research report on the application of AI in pyloric obstruction.

#### Cancerization

DU rarely become cancerous, while GU may become cancerous, especially in those with *Hp* infection. More than 70% of early gastric cancer (EGC) patients have no obvious symptoms (35). As the disease progresses, symptoms of gastritis or gastric ulcer may gradually appear, including loss of appetite, nausea, indigestion, weight loss, upper abdominal discomfort or dull pain, and occasionally vomiting, fecal occult blood or melena, pantothenic acid deficiency, unexplained fatigue or progressive anemia. As an important basis for choosing treatment options for EGC, tumor classification and microscopic staging are very important. Esophagogastroduodenoscopy (EGD) biopsy is considered the current standard method for identifying gastric mucosal lesions. Endoscopists must have considerable experience and knowledge to correctly diagnose malignant ulcers, but this often requires long-term technical training and experience accumulation. Machines have fewer variations within and between observers, and the results are generally better than those of human endoscopists (36). The advancement of AI technology can provide higher sensitivity and specificity for the recognition and diagnosis of EGC under endoscopy.

The work of Ken Namikawa et al. proved that after training the AI-based diagnostic system with a large amount of data, the diagnostic accuracy of gastric cancer and GU classification reached a very high level, with a comprehensive diagnostic accuracy of 95.9% (37). Recently, E. Klang et al. built a CNN model aimed at distinguishing benign and malignant GU from endoscopic images in the western population (38). The study retrospectively collected endoscopic images of benign and malignant GU patients undergoing endoscopy at the Chaim Sheba Medical Center from 2011 to 2019. Every included image had a corresponding biopsy result that was sampled at the same time as endoscopic examination. Endoscope images from 2011 to 2015 and from 2016 to 2017 were used for training and validation, while the retained data from 2018 to 2019 were used to test the final model. In addition, some public pictures were obtained through the Google image search engine for pretraining the model. The final model showed an AUC of 0.91 to detect malignant ulcers. For a cut-off probability of 0.5, the model had a sensitivity of 92% and a specificity of 75%.

Moreover, a DL model based on endoscopic images to diagnose gastric mucosal lesions was developed by Joon Yeul Nam et al. (39). This model was based on a CNN algorithm to achieve the purpose of lesion detection, differential diagnosis (AI-DDx model), and depth of invasion detection (AI-ID model). A total of 1,366 patients from 2 referral centers with gastric mucosal lesions were consecutively included in this study. Representative endoscopic images of benign GU, EGC or advanced gastric cancer selected by experts for each patient were used as the training and testing sets, with the histological diagnoses as the gold standard. The results identified by the models were compared with the visual diagnosis and ultrasound endoscopy results of endoscopists with different working years. The results showed that the AI-DDx model performed better than novice and intermediate endoscopists, was comparable to expert endoscopists, and reached AUCs of 0.86 in both internal and external validation. The AUCs of the AI-ID model were 0.78 in the internal validation and 0.73 in the external validation, which were significantly better than the endoscopic ultrasonography results performed by experts. In general, there are numerous related studies on the application of AI in GU complications (40–43), and detailed information can be found in Table 1.

**Table 1:** Summary of applications of AI in PU’s complications.

Ref.	Year	AI technology	Research Objectives	Training and Validating set	Outcomes
Karakitsos et al. (40)	1998	ANN	To discriminate benign and malignant gastric cells	2,500 cells from 23 cancer, 19 gastritis and 58 ulcer cases for training, 8,524 cells from the same cases for testing	Correct classification of >97% benign cells and >95% malignant cells, overall accuracy of >97%
Grossi et al. (41)	2008	ANN	To recognize patients at high risk of death for nonvariceal upper GI bleeding	807 patients with nonvariceal upper GI bleeding	Average sensitivity of 89.2%, average specificity of 82.9%, average accuracy of 86%, and AUC of 0.87
Rotondano et al. (42)	2011	ANN	To predict mortality in patients with nonvariceal upper GI bleeding	2,380 patients with nonvariceal upper GI bleeding	Sensitivity of 83.8%, specificity of 97.5%, accuracy of 96.8%, and AUC of 0.95
Søreide et al. (33)	2015	ANN	To predict outcomes in patients with perforated gastroduodenal ulcers	117 patients for training and 51 patients for testing	AUC of inclusive, multifactorial ANN model is 0.90
Tan et al. (30)	2018	Deep residual network	To predict PU bleeding mortality	6,367 patients diagnosed with PU bleeding	AUC of 0.94 for PU bleeding mortality prediction
Wong et al. (31)	2019	ML	To identify patients at high risk for recurrent ulcer bleeding	22 854 patients with PU for training and 1,265 patients with PU for testing	Overall accuracy of 84.3% and AUC of 0.78
Lee et al. (43)	2019	Deep neural network and transfer-learning approach	To discriminate benign ulcer and cancer	180 normal, 200 benign ulcer, and 337 cancer images for training and 20, 30, 20 images for testing	Accuracies of discriminating Normal vs cancer, Normal vs ulcer, and Cancer vs ulcer were 96.5%, 92.6% and 77.1%
Nakashima et al. (37)	2020	Advanced CNN	To discriminate gastric cancers and gastric ulcers	13,584 gastric cancer and 4,826 gastric ulcer images for training, 739 gastric cancer images and 720 gastric ulcer images for validation	Sensitivity of 93,3%, specificity of 99.0% and positive predictive value of 99.1% for gastric ulcer
Tan et al. (29)	2021	A novel end-to-end importance perception personalized deep learning method	To predict bleeding risk	6,367 patients with peptic ulcer bleeding	AUC of 0.944 at 1 year ahead of risk prediction
Klang et al. (38)	2021	CNN	To discriminate benign and malignant GU	1,299 images for training, 364 images for validation and 315 images for testing	Sensitivity of 92%, specificity of 75% and AUC of 0.91 for detecting malignant ulcers
Nam et al. (39)	2021	AI-differential diagnosis	To diagnose gastric mucosal lesions (GU, EGC, AGC)	1,009 patients for training, 112 patients for internal validation and 245 patients for external validation	AUC of 0.86 in diagnostic performance for both internal and external validation

*AI, Artificial intelligence; PU, Peptic Ulcer; ANN, Artificial Neural Networks; CNN, Convolutional Neural Network; GI, Gastrointestinal; DL, Deep Learning; EGC, Early Gastric Cancer; AGC, Advanced Gastric Cancer; AUC, Area Under the Curve; Hp, Helicobacter pylori.*

## Challenges and Prospects

Although significant progress has been made in the application of AI in the diagnosis, management and complication prediction of PU, there are still the following areas for improvement. First, the majority of related studies have a small sample size, and only include high-quality images for AI modeling, which cannot reflect the differences in inspection equipment covering hospitals at all levels and complex clinical scenarios. Second, most studies related to AI and GU are retrospective studies, which may overestimate the real performance of AI models due to selection bias. Moreover, few studies have evaluated the auxiliary role of AI systems for endoscopists, especially juniors. Finally, most studies only consider a certain aspect of clinical information such as imaging or biomarkers, and the integration of clinical multimodal data seems essential to further improve the performance of AI systems.

Despite the fact that AI has made major breakthroughs in the field of PU, we have more expectations for the optimization of AI systems. For instance, how can AI algorithms suitable for different scenarios be developed to achieve robust, versatile and diverse multifunctional AI systems in the PU field? How can a multifunctional AI system be integrated in the future to realize the whole process management of PU lesions from risk assessment and diagnosis to treatment? Furthermore, with the gradual improvement of residents’ health awareness, an increasing number of people take health check-ups. It is hoped that in the future, different endoscopic images from healthy mucosa to severe PU can be collected to study mucosal and microvascular changes before lesions occur to identify GU lesions at an early stage. Finally, and most importantly, it is urgent to carry out multicenter and large-sample clinical research on the application of AI in PU, hoping to provide a solid theoretical basis for the transformation and application of AI systems (Supplementary Figure S1).

## Conclusion

This minireview elaborates on the research progress of AI in the field of PU, from PU’s pathogenic factor *Helicobacter pylori (Hp)* infection, diagnosis and differential diagnosis, to its management and complications (bleeding, obstruction, perforation and canceration). Finally, the challenges and prospects of AI application in PU are prospected and expounded. It provides us with an in-depth understanding of the research status of AI in the PU field. Numerous preclinical and clinical studies have clearly demonstrated the feasibility and safety of AI, which not only ensures the diagnostic accuracy but also greatly improves diagnostic efficiency. AI unquestionably makes a significant contribution to reducing the workload of gastrointestinal endoscopists. At the same time, AI still has some limitations in the field of PU, such as an insufficient research sample size, and existing conclusions are mainly based on retrospective research. How to realize the robustness, versatility and diversity of multifunctional AI systems in PU and conduct multicenter prospective clinical research as soon as possible are the top priorities in the future.

## Author Contributions

P-yZ and KH performed the majority of the writing, prepared the figures and tables. R-qY and CR edited and revised the manuscript. X-hD designed the study; all authors approved the final version to be published. All authors contributed to the article and approved the submitted version.
