# Supplementary figures and images for: Application Status and Prospects of Artificial Intelligence in Peptic Ulcers
Source: Front Surg. 2022 Jun 16;9:894775. doi: 10.3389/fsurg.2022.894775 (PMC9244632; doi:10.3389/fsurg.2022.894775)

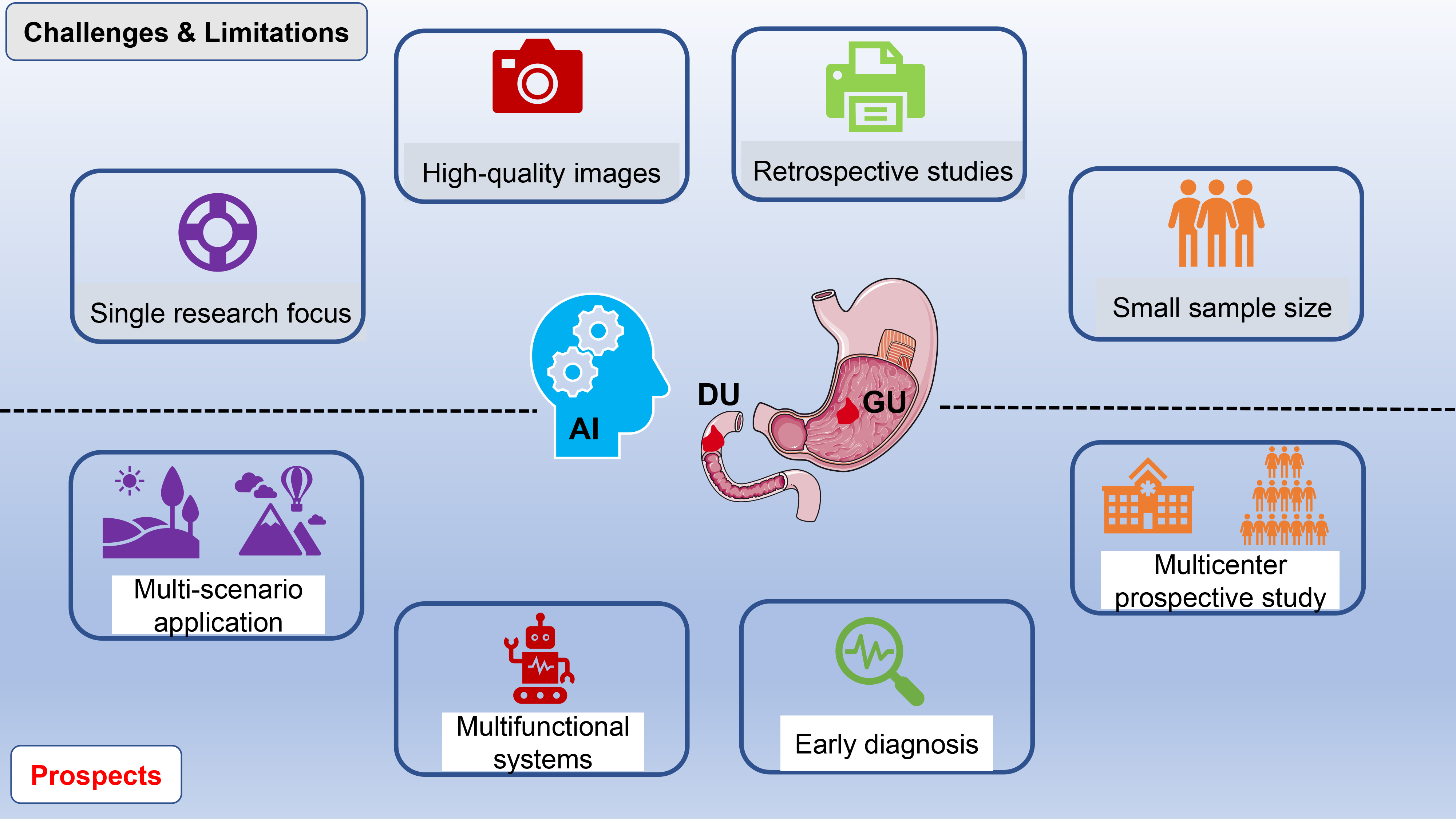

Supplement: Supplementary file 1 [file Image_1_v1.tif]
