# Supplementary material for: Application Status and Prospects of Artificial Intelligence in Peptic Ulcers
Source: Front Surg. 2022 Jun 16;9:894775. doi: 10.3389/fsurg.2022.894775 (PMC9244632; doi:10.3389/fsurg.2022.894775)
Supplement: Supplementary file 2 [file Table_1_v1.docx]

| **Supplemental Table S1 Summary of applications of AI in predicting *Hp* infection.** | | | | | |  |
| --- | --- | --- | --- | --- | --- | --- |
| **Ref.** | **Year** | **AI technology** | **Training set** | **Validating set** | **Outcomes** | |
| Huang et al [10] | 2004 | Computer-assisted image-analyzing system | 30 patients (15 with and 15 without *Hp* infection) | 74 patients | Sensitivity of 85.4% and specificity of 90.9% | |
| Shichijo et al [11] | 2017 | Deep CNN | 32,208 images from 1750 patients (735 with and 1015 without *Hp* infection) | 11,481 images from 397 patients (72 with and 325  without *Hp* infection) | Sensitivity of 88.9%, specificity of 87.4%, accuracy of 87.7%, and diagnostic time of 194s | |
| Itoh et al [12] | 2018 | CNN | 149 images from 139 patients (65 with and 74 without *Hp* infection) | 30 images (15 with and 15 without *Hp* infection) | Sensitivity of 86.7%, specificity of 86.7% and AUC of 0.956 | |
| Nakashima et al [13] | 2018 | DL | 1944 images from 162 patients (75 with and 87 without *Hp* infection) | 180 images from 60 patients (30 with and 30 without *Hp* infection) | Sensitivity of 96.7%, specificity of 86.7% and AUC of 0.96 in BLI-bright; Sensitivity of 96.7%, specificity of 83.3% and AUC of 0.95 in LCI | |
| Shichijo et al [14] | 2019 | Deep CNN | 98564 images from 5236 patients (742 with and 3649 without *Hp* infection, 845 post-eradication status) | 23699 images (418 with and 23034 without *Hp* infection, 247 post-eradication status) | Accuracy of *Hp* negative diagnosis, positive diagnosis and eradicated diagnosis were 80%, 48% and 84%. The total time was 261 seconds. | |
| Zheng et al [15] | 2019 | CNN | 11729 images from 1507 patients (847 with and 660 without *Hp* infection) | 3755 images from 452 patients (310 with and 142 without *Hp* infection) | Sensitivity of 81.4%, specificity of 90.1%, accuracy of 84.5% | |
| Nakashima et al [16] | 2020 | computer-aided  diagnosis (CAD) | 12887 images from 395  Patients (138 with and 141 without *Hp* infection, 116 post-eradication status) | 240 images from 120  Patients (40 with and 40 without *Hp* infection, 40 post-eradication status) | Sensitivity of 62.5%, specificity of 92.5% and AUC of 0.82 | |

Hp: Helicobacter pylori; AI: Artificial intelligence; CNN: Convolutional Neural Network; DL: Deep Learning; CAD: Computer Aided Diagnosis; AUC: Area Under the Curve; BLI: Blue Laser Imaging; LCI: Linked Color Imaging.

| **Supplemental Table S2. Summary of applications of AI in diagnosing PU** | | | | | |  |
| --- | --- | --- | --- | --- | --- | --- |
| **Ref.** | **Year** | **AI technology** | **Research Objectives** | **Training and Validating set** | **Outcomes** | |
| Sáenz Bajo et al [17] | 2002 | Neurone network | to discriminate PU and functional or idiopathic dyspepsia | 81 patients with a diagnosis of  dyspepsia | classifying 81% of patients, with negative  predictor value of 90% and positive predictor  value of 80% | |
| Al-Kasasbeh et al [18] | 2013 | Fuzzy logic  decision-making | To solve the problem of prediction of gastric ulcer | none | the level of errors in prediction is  not higher than 0.18 | |
| Wang et al [19] | 2019 | Deep CNN | To detect ulcer in WCE | 15781 ulcer frames for training, 2040 ulcer and 2319 normal frames for validation, 4917 ulcer and 5007 normal frames for testing | Overall sensitivity of 89.7%, overall specificity of 90.5% and overall accuracy of 90.1% | |
| Alaskar et al [20] | 2019 | CNN | To detect gastrointestinal ulcer | 336 images for training, 105  images for testing | Sensitivity of 100%, specificity of 100% and accuracy of 100% | |
| Wang et al [24] | 2019 | Deep CNN | To recognize ulcer in WCE | 1416 independent WCE videos (1157 ulcer and 259 normal) | overall test accuracy of 92.05%, sensitivity of 91.64% and specificity of 92.42% | |
| Khan et al [21] | 2019 | Rank-Based Deep  Features Selection | To recognize stomach deformities | 12000 video frames (4000 ulcer, 4000 bleeding and 4000 normal) for validating | accuracy of 99.5% and total time  of 21.15 s | |
| Majid et al [22] | 2020 | CNN | To detect and classify  gastric infections | 9,889 images of four stomach infections | accuracy of 96.5% | |
| Xia et al [23] | 2021 | CNN | To detect gastric lesions | 1023955 images from 797 patients | Sensitivity of 96.2%, specificity of 76.2%, accuracy of 77.1%, and AUC of 0.84 | |
| Guo et al [25] | 2021 | CAD | to detect the four categories of gastrointestinal lesions | 327121 images from 117005 cases for training and 33959 images from 1734 cases  for validation | Sensitivity of 92.4%, specificity of 90.3% and detect efficiency of 89.7% for detecting erosion/ulcer | |
| Zhang et al [26] | 2021 | Deep-CNN | To diagnose five  gastric conditions | 21217 images of PU, EGC and HGIN, AGC, SMTs  and normal | Sensitivity of 71.1%, specificity of 87.3% and accuracy of 83.9% for PU | |

AI: Artificial intelligence; PU: Peptic Ulcer; CNN: Convolutional Neural Network; WCE: Wireless Capsule Endoscopy; CAD: Computer Aided Diagnosis; AUC: Area Under the Curve; EGC: Early Gastric Cancer; HGIN: High-grade Intraepithelial Neoplasia; AGC: Advanced Gastric Cancer; SMT: Gastric Submucosal Tumors.
